# Cardiopulmonary bypass increases the risk of vasoplegic syndrome after coronary artery bypass grafting in patients with dialysis-dependent chronic renal failure

**DOI:** 10.5935/1678-9741.20140092

**Published:** 2015

**Authors:** Nelson Américo Hossne Junior, Matheus Miranda, Marcus Rodrigo Monteiro, João Nelson Rodrigues Branco, Guilherme Flora Vargas, José Osmar Medina de Abreu Pestana, Walter José Gomes

**Affiliations:** 1 Escola Paulista de Medicina da Universidade Federal de São Paulo (EPMUNIFESP), São Paulo, SP, Brazil.

**Keywords:** Myocardial Revascularization, Renal Insufficiency, Chronic, Vasoplegic Syndrome, Cardiopulmonary Bypass

## Abstract

**Objective:**

Coronary artery bypass grafting is currently the best treatment for dialysis patients with multivessel coronary artery involvement. Vasoplegic syndrome of inflammatory etiology constitutes an important postoperative complication, with highly negative impact on prognosis. Considering that these patients have an intrinsic inflammatory response exacerbation, our goal was to evaluate the incidence and mortality of vasoplegic syndrome after myocardial revascularization in this group.

**Methods:**

A retrospective, single-center study of 50 consecutive and non-selected dialysis patients who underwent myocardial revascularization in a tertiary university hospital, from 2007 to 2012. The patients were divided into 2 groups, according to the use of cardiopulmonary bypass or not (off-pump coronary artery bypass). The incidence and mortality of vasoplegic syndrome were analyzed. The subgroup of vasoplegic patients was studied separately.

**Results:**

There were no preoperative demographic differences between the cardiopulmonary bypass (n=20) and off-pump coronary artery bypass (n=30) group. Intraoperative data showed a greater number of distal coronary arteries anastomosis (2.8 *vs.* 1.8, *P*<0.0001) and higher transfusion rates (65% *vs.* 23%, *P*=0.008) in the cardiopulmonary bypass group. Vasoplegia incidence was statistically higher (*P*=0.0124) in the cardiopulmonary bypass group (30%) compared to the off-pump coronary artery bypass group (3%). Vasoplegia mortality was 50% in the cardiopulmonary bypass group and 0% in the off-pump coronary artery bypass group. The vasoplegic subgroup analysis showed no statistically significant clinical differences.

**Conclusion:**

Cardiopulmonary bypass increased the risk for developing postoperative vasoplegic syndrome after coronary artery bypass grafting in patients with dialysis-dependent chronic renal failure.

## Abstract (pt)

**Objetivo:**

O melhor tratamento atual para os pacientes dialíticos com acometimento coronariano multiarterial é a revascularização cirúrgica do miocárdio. Dentre as complicações pós-operatórias, a síndrome vasoplégica de etiopatogenia inflamatória torna-se importante pelo impacto altamente negativo no prognóstico. Considerando que esses pacientes possuem uma exacerbação intrínseca da resposta inflamatória, nosso objetivo foi avaliar a incidência e a mortalidade da síndrome vasoplégica no pós-operatório de revascularização miocárdica nesse grupo.

**Métodos:**

Estudo retrospectivo, unicêntrico, de 50 pacientes dialíticos consecutivos e não selecionados, submetidos à revascularização miocárdica, em um hospital terciário universitário, no período de 2007 a 2012. Esses pacientes foram divididos em 2 grupos, de acordo com o emprego ou não da circulação extracorpórea. A incidência e a mortalidade da vasoplegia foram analisadas nos grupos. Após a identificação dos pacientes quanto à presença de vasoplegia, este subgrupo foi estudado separadamente.

**Resultados:**

Não houve diferenças demográficas pré-operatórias entre os grupos com circulação extracorpórea (n=20) e sem circulação extracorpórea (n=30). Dados intraoperatórios demonstraram maior número de artérias coronárias revascularizadas (2,8 *vs.* 1,8; *P*<0,0001) e maior necessidade de transfusão (65% *vs.* 23%; *P*=0,008) no grupo circulação extracorpórea. A incidência de vasoplegia foi estatisticamente maior (*P*=0,0124) no grupo circulação extracorpórea (30%) em comparação ao grupo sem circulação extracorpórea (3%). A mortalidade dos pacientes com vasoplegia foi 50% no grupo circulação extracorpórea e 0% no grupo sem circulação extracorpórea. A análise do subgrupo vasoplégico não demonstrou diferenças clínicas estatisticamente significantes.

**Conclusão:**

O emprego da circulação extracorpórea na revascularização cirúrgica do miocárdio em pacientes com insuficiência renal crônica dialítica aumentou o risco para desenvolvimento de síndrome vasoplégica pós-operatória.

**Table:** 

**Abbreviations, acronyms & symbols**	
ACE	Angiotensin-converting enzyme
ACT	Activated clotting time
CABG	Coronary artery bypass grafting
CI	Cardiac index
COPD	Chronic obstructive pulmonary disease
CPB	Cardiopulmonary bypass
CRF	Chronic renal failure
LV	Left ventricle
MAP	Mean arterial pressure
OPCAB	Off-pump coronary artery bypass.
SVRI	Systemic vascular resistance index

## INTRODUCTION

Chronic renal failure (CRF) constitutes an independent risk factor for chronic coronary artery disease, and the severity of coronary lesions are inversely proportional to glomerular filtration rate^[1]^. Several patients' characteristics are associated with a greater severity of coronary disease in this group, such as uremia, poor quality of distal coronary bed, hyperhomocysteinemia, increased calcium-phosphorus product, oxidative stress, among others. Additionally, the chronic inflammatory status enhanced by CRF contributes to amplify the already established inflammatory pathogenesis of coronary atherosclerosis^[2]^.

Thus, ischemic cardiovascular diseases are the leading cause of mortality in this class of patients^[1]^.

Coronary artery bypass grafting (CABG) shows greater long-term survival and lower risk of myocardial infarction and death from cardiovascular events compared to coronary angioplasty and stenting in patients with chronic renal failure requiring dialysis therapy^[3]^. However, this group of patients experiences high morbidity and mortality mainly due to the presence of multiple preoperative comorbidities^[4]^. Furthermore, several reports have shown greater inflammatory response in patients undergoing cardiovascular surgery with cardiopulmonary bypass (CPB) compared to off-pump surgery^[5,6]^.

International guidelines recommend performing coronary artery bypass surgery without the use of CPB in dialysis patients, whenever possible, since its use can lead to increased postoperative morbidity^[7,8]^.

Vasoplegic syndrome is a well-recognized complication in the postoperative setting of cardiovascular surgery, initially described by Gomes et al.^[9,10]^. It can be defined as a hemodynamic shock, resembling septic shock syndrome, in which there is evidence of decreased systemic vascular resistance index, increased cardiac index and severe hypotension with the use of vasoactive drugs, initiating in the early postoperative hours. Although the mechanisms are not fully understood, most authors propose a direct correlation between the release of inflammatory mediators and severe vasodilation with consequent vasoplegic syndrome^[11-13]^. Its incidence varies widely among several reports, between 5% and 44%, with median values of 10%. Higher incidence values are generally found in groups considered at high risk of developing vasoplegia, such as patients with left ventricular assistance, ventricular dysfunction, preoperative use of angiotensin converting enzyme inhibitors and heparin, and other factors with discordant correlation reported^[11,14-16]^.

Considering the well-established inflammatory pathogenesis of coronary atherosclerosis, the inherent chronic inflammatory status presented in patients with CRF requiring dialysis^[17]^, the greater inflammatory response in patients undergoing CPB, as well as the inflammatory mediators intrinsically linked to severe vasodilation of the vasoplegic syndrome, we would expect a higher incidence of vasoplegia in dialysis patients undergoing CABG with CPB.

Therefore, our objective was to analyze the incidence and mortality of vasoplegic syndrome in patients with CRF/dialysis who underwent CABG with and without the use of CPB.

## METHODS

This was a single-center, retrospective study of 50 consecutive patients with chronic renal failure on dialysis referred for CABG at a tertiary public university hospital, from 2007 to 2012. Patients with concomitant surgical procedures (valve surgery, carotid, aortic, etc.) were excluded from the study.

Patients' demographic and clinical characteristics, intraoperative data and postoperative complications during hospitalization were evaluated, as well as the incidence and mortality of postoperative vasoplegic syndrome. Vasoplegic syndrome was defined by hypotension (MAP<60 mmHg) refractory to vasopressor drugs administration (norepinephrine, epinephrine and vasopressin), decreased systemic vascular resistance index (SVRI<1.600 dyn·sec/cm^5^/m^2^) and high cardiac index (CI>2.5 L/min/m^2^), measured by continuous thermodilution catheter.

The European System for Cardiac Operative Risk Evaluation II (EuroSCORE II) was used for preoperative risk calculation^[18]^.

Patient sample was divided into two groups, according to the use of CPB, in order to identify variables that implied higher morbidity and in-hospital mortality, with detailed specific analysis in the subgroup of patients with vasoplegia. The study was approved by the Institutional Ethics Committee.

### Surgical technique for coronary artery bypass grafting

Surgical referral for myocardial revascularization was based on American and European guidelines^[15,16]^, with all patients with class I recommendation - obstructive lesions greater than 50% in the left main coronary or greater than 70% in patients with multivessel and proximal left anterior descending disease.

All patients underwent hemodialysis with heparin and subsequent reversal with protamine the day before the surgical procedure.

Preoperative planning was done according to the severity of the coronary lesions and possibility of surgical revascularization of the distal coronary bed. Surgical technique was standardized with the left internal thoracic artery for revascularization of the anterior descending artery, and saphenous vein used for other coronary beds, with proximal anastomosis in the ascending aorta.

All patients were operated through median sternotomy. Dissection of the left internal thoracic artery was performed very carefully, avoiding the left pleura space opening. The saphenous vein was removed through small incisions on the thigh.

The use of cardiopulmonary bypass or off-pump technique was based on the main surgeon judgment criteria, according to each patient characteristics.

### Technique with cardiopulmonary bypass

CPB establishment (Braile Biomédica, Sao José do Rio Preto, São Paulo, Brazil), including arterial and venous cannulas, consisted of a standard circuit with cardiotomy reservoir, membrane oxygenator, roller pump, and arterial line filter.

Heparin (Blausiegel, Blau, São Paulo, Brazil) was administered before aortic and atrial cannulation (dual-stage cannula) at a dose of 4 mg/kg, directly in the right atrium. Cardiopulmonary bypass with mild systemic hypothermia (32º C), at a rate of 1.8 L/m^2^/ min to maintain MAP>60 mmHg, was initiated only after activated coagulation time (ACT) (MCA-2000, Adib Jatene Foundation, São Paulo, Brazil) above 480 seconds, ten minutes after heparin administration. Noradrenaline was administered for patients with MAP < 60 mmHg during CPB, despite adequate blood volume and flow. Heparin dosage and efficacy during CPB was controlled by sequential ACT measurements, every 30 minutes, and supplemented with 1 mg/kg heparin whenever ACT < 480 seconds. Myocardial protection with anterograde hypothermic (4ºC) blood cardioplegia (25% hematocrit) was intermittently performed every 15 minutes through aortic puncture, with 400 mL/min flow (MAP > 60 mmHg), and pulmonary artery trunk continuous aspiration (left ventricle drainage) during cardiac arrest with continuous aortic cross clamping.

### Off-Pump Technique

Heparin (Blausiegel, Blau, São Paulo, Brazil) was administered 10 minutes prior to coronary occlusion at a 2 mg/kg dose. Distal anastomoses were performed employing a suction stabilizer (Octopus, Medtronic, Inc., Minneapolis, MN, United States), with only proximal tourniquet of coronary arteries with Polypropylene 5-0 (Prolene, Johnson & Johnson, New Jersey, NY, United States). Coronary anastomoses of totally occluded vessels were prioritized.

Heparin reversal was performed at the end of the surgery for all patients, with protamine at a 1:1 dose, through a peripheral intravenous route, regardless of the surgical technique.

### Statistical analysis

Chi-square and Fisher exact tests were used for qualitative variables to compare the groups according to the use of CPB. Mann-Whitney test was employed for quantitative variables. These same tests were also used for comparison between subsets of vasoplegic patients.

Statistical significance was set at 0.05 or 5%.

Statistical tests were calculated with the BioEstat 5.0 software.

## RESULTS

Demographic data of all patients are shown in Table 1.

**Table 1:** Demographic characteristics, according to the use of cardiopulmonary bypass.

Demographic Characteristics	CPB	Off-pump	*P* value
n	20	30	0.68
Age (years)	57.1	56.1	0.96
Female gender (%)	20	37	0.34
Dialysis time (months)	3.5	5.0	0.68
Hypertension (%)	100	100	1.00
Diabetes (%)	70	72.6	0.95
Dyslipidemia (%)	50	37	0.52
Obesity (%)	5	20	0.22
Smoking (%)	15	7	0.38
COPD(%)	0	0	1.00
Previous stroke (%)	15	17	1.00
Previous coronary stenting (%)	25	27	0.84
Previous cardiac surgery (%)	0	0	1.00
Congestive heart failure (%)	25	23	0.81
Previous myocardial infarction (%)	15	20	0.72
Peripheral artery disease (%)	15	20	0.72
Stable angina (%)	10	17	0.69
Unstable angina (%)	15	13	0.99
Left main disease (%)	15	17	1.00
LV ejection fraction > 50%	80	77	1.00
EuroSCORE II	2.5	2.8	0.83

COPD=chronic obstructive pulmonary disease; CPB=cardiopulmonary bypass; LV=left ventricle; Left main disease=obstructive lesion greater than 50%

Sixteen percent of patients had left main coronary artery lesions (greater than 50% obstruction). The remaining patients (84%) had multivessel disease.

Three patients were in immunosuppressive therapy due to prior renal transplant; however, they returned to dialysis therapy after kidney graft rejection.

No patients had preoperative hemodynamic instability nor were they referred for urgent or emergency surgery.

Intraoperative variables are presented in Table 2. There were no intraoperative deaths.

**Table 2:** Intraoperative variables, according to the use ofcardiopulmonary bypass.

Intraoperative variable	CPB	Off-pump	*P* value
Number of coronary anastomosis	2.8	1.8	<0.0001
Inotropic support (%)	85	40	1.00
Blood transfusion (%)	65	23	0.008

Vasoplegic syndrome incidence was 30% in the CPB group and 3% in the off-pump group, as shown in Table 3.

**Table 3:** Incidence of postoperative vasoplegia, according to the use cardiopulmonary bypass.

Vasoplegia	CPB	Off-pump	*P* value
Incidence (%)	30	3	0.01

The analysis of clinical characteristics of the vasoplegic patients, according to the use of cardiopulmonary bypass, is described in Table 4.

**Table 4:** Vasoplegic subgroup characteristics, according to the use of cardiopulmonary bypass.

	Vasoplegic subgroup	CPB	Off-pump	*P* value
n		6	1	0.01
	AAS	100%	100%	1.00
Preoperative	Beta-blocker	83.3%	100%	1.00
medication	ACE inhibitor	16.7%	0%	1.00
	Statin	100%	100%	1.00
LV ejection fraction > 50%		100%	100%	1.00
Diabetes (%)		66.7%	100%	1.00

The mean ± standard deviation of bypass and aortic cross clamp times in the CPB patients who developed vasoplegia compared to patients who did not develop vasoplegia in the same group are shown in Table 5.

**Table 5:** Average ± standard deviation (in minutes) of bypass and aortic cross clamp times, according to the development of postoperative vasoplegia.

Mean time ± standard deviation	Vasoplegia	No vasoplegia	*P* value
Bypass	102.7±20.5	81.3±29.3	0.08
Aortic cross clamp	58.5±16.6	56.3±21.0	0.90

Vasoplegic syndrome mortality was 50% (3/6) in the CPB subgroup. There were no deaths in the off-pump group (0/1).

## DISCUSSION

The high prevalence of cardiovascular risk factors in patients with chronic renal failure in dialysis therapy in this study reiterates ischemic cardiovascular disease as the leading cause of mortality in this group of patients.

Considering our consecutive series, 52% of patients had no cardiovascular symptoms, and 92% presented with preserved left ventricular function. It is noteworthy that most patients were referred for CABG after the diagnosis of severe coronary lesions in elective coronary angiography protocol for kidney pre-transplant evaluation; particularly, if we consider that CABG reduces kidney transplant operative risk, and increases the immediate survival of these patients^[19]^.

Group pairing comparison, according to the use of CPB, demonstrated no statistical differences in relation to preoperative demographic data or surgical risk, even in a retrospective analysis.

Intraoperative data showed a higher mean number of distal anastomosis in the CPB group compared to the off-pump group. The bad quality of the thin distal coronary bed in some patients precluded a complete revascularization, despite the typical multivessel involvement usually present in dialysis patients. The criteria on whether to use CPB were homogeneous since the same surgical team performed all surgeries.

It is important to point out that the diagnosis of vasoplegic syndrome was conducted in an objective manner, with assessment of all hemodynamic measurements through continuous thermodilution catheter, defined by hypotension (MAP<60 mmHg) refractory to vasopressor drugs administration (norepinephrine, epinephrine and vasopressin), decreased systemic vascular resistance index (SVRI<1.600 dyn·sec/cm^5^/m^2^), and high cardiac index (CI > 2.5 L/min/m^2^).

Overall incidence of vasoplegia in our study was 12%. However, a higher incidence (*P*=0.01) of vasoplegic syndrome was observed in patients with chronic renal failure/dialysis who underwent on-pump CABG surgery (30%) compared to the off-pump group (3%).

Analysis of the vasoplegic subgroup did not show any statistically significant differences in relation to clinical characteristics, according to the use of CPB. Published data had shown that preoperative use of ACE inhibitors has been associated with an increased risk of vasoplegic syndrome^[16]^. Nevertheless, no differences were detected in the use of preoperative medications, including ACE inhibitors, beta blockers, aspirin or statins in the vasoplegic subgroup.

Cardiopulmonary bypass time in patients who developed vasoplegia was slightly higher than in patients without vasoplegic syndrome, within the CPB group (*P*=0.08). However, evidence supports a direct correlation between longer CPB times and the risk of developing vasoplegic syndrome^[11,14,20]^.

Transfusion of blood products was higher in the CPB group. Despite the well-established exacerbation of postoperative inflammatory response in patients undergoing blood transfusions, available data regarding the correlation between the number of transfused blood products and incidence of postoperative vasoplegic syndrome is conflicting^[11,12,14,15,20]^.

Current risk factors for the development of postoperative vasoplegia have not been fully elucidated. Most authors seem to agree that preoperative use of ACE inhibitors, or heparin, increases the risk of vasoplegia^[16]^. Other preoperative factors with conflicting data are the use of beta-blockers, diabetes, and severe left ventricular dysfunction^[11-15,20,21]^. Surgical trauma itself may be responsible for some reported series of vasoplegia after off-pump cardiovascular surgeries^[22]^.

Treatment of patients with vasoplegic syndrome in our study consisted of vasopressor support (norepinephrine, epinephrine and vasopressin) and methylene blue^[12,16,23]^. The only patient with vasoplegia in the off-pump group showed gradual improvement, with recovery of severe vasodilation within 36 hours.

Overall mortality was high in the vasoplegic patients (42.86%), accounting for three of the seven in-hospital deaths in our study. Specific mortality was 50% for the CPB vasoplegic subgroup, and 0% for the off-pump vasoplegic subgroup. Our vasoplegic syndrome mortality was higher than the 25% rate usually published for patients with severe refractory vasodilation for more than 48 hours^[12,13]^.

The pathophysiological mechanisms of vasoplegic syndrome remain controversial; however, there is clear association between pro-inflammatory mediators' production and subsequent induction of nitric oxide production and GMPc mediated severe vasodilation. The rational use of vasopressin and methylene blue is based upon nitric oxide inhibition. Nevertheless, other pathways not directly related to nitric oxide release have been described, such as the direct activation of the final common pathway of vasodilation through guanylate cyclase enzyme, mediated by the release of pro-inflammatory molecules after CPB^[11-13,24]^. This pathway may explain the inconsistent reports of decreased mortality in vasoplegic syndrome with the use of vasopressin and methylene blue^[16,23,25]^.

Considering the available data, an increased inflammatory response is clearly acting as a common pathophysiological role for the development of vasoplegic syndrome after cardiac surgery. The well described inflammatory reaction after the use of CPB coupled with the intrinsic status of chronic inflammation in patients with CRF, particularly hemodialysis, suggest that this combination of factors may complete the *inflammatory equation* that could explain a higher incidence of vasoplegia observed in the group of patients who underwent CPB in our data.

Thus, our results support the current guidelines recommendation to perform off-pump CABG in patients with chronic renal failure requiring dialytic therapy.

### Study limitations

We performed a retrospective analysis with a relatively small number of patients. Moreover, the common exclusion of these patients from larger trials may also interfere in the choice of the best surgical technique and results.

Prospective studies with larger numbers of patients are needed to support our results.

## CONCLUSION

Coronary artery bypass grafting with cardiopulmonary bypass in patients with chronic renal failure requiring dialysis therapy was an independent risk factor for the development of vasoplegic postoperative syndrome.

Off-pump coronary artery bypass surgery seems to be a friendly alternative for the treatment of these patients.

**Table:** 

**Authors’ roles & responsibilities**	
NAHJ	Analysis and/or data interpretation; conception and design study; final manuscript approval; manuscript writing or critical review of its content; conduct of operations and/or trials; statistical analysis
MM	Analysis and/or data interpretation; conception and design study; final manuscript approval; manuscript writing or critical review of its content; statistical analysis
MRM	Analysis and/or data interpretation, conception and design study; final manuscript approval; manuscript writing or critical review of its content
JNRB	Analysis and/or data interpretation; conception and design study; final manuscript approval; manuscript writing or critical review of its content; conduct of operations and/or trials; statistical analysis
GFV	Final manuscript approval; manuscript writing or critical review of its content; conduct of operations and/or trials
JOMAP	Analysis and/or data interpretation; conception and design study; final manuscript approval; manuscript writing or critical review of its content
WJG	Analysis and/or data interpretation; final manuscript approv-al; manuscript writing or critical review of its content
